# Neovascularization of hepatocellular carcinoma in a nude mouse orthotopic liver cancer model: a morphological study using X-ray in-line phase-contrast imaging

**DOI:** 10.1186/s12885-017-3073-3

**Published:** 2017-01-25

**Authors:** Beilei Li, Yiqiu Zhang, Weizhong Wu, Guohao Du, Liang Cai, Hongcheng Shi, Shaoliang Chen

**Affiliations:** 1 Department of Nuclear Medicine, Zhongshan Hospital, Fudan University, No.180, Fenglin Road, Shanghai, 200032 China; 2 Shanghai Institute of Medical Imaging, Shanghai, 200032 China; 3 Liver Cancer Institute, Zhongshan Hospital, Fudan University, Shanghai, 200032 China; 4 Shanghai Institute of Applied Physics, Chinese Academy of Sciences, Shanghai, 201800 China

**Keywords:** Synchrotron radiation, In-line phase-contrast imaging, Computed tomography, Hepatocellular carcinoma, Tumor neovascularization

## Abstract

**Background:**

This study aimed to determine whether synchrotron radiation (SR)-based X-ray in-line phase-contrast imaging (IL-PCI) can be used to investigate the morphological characteristics of tumor neovascularization in a liver xenograft animal model.

**Methods:**

A human hepatocellular carcinoma HCCLM3 xenograft model was established in nude mice. Xenografts were sampled each week for 4 weeks and fixed to analyze tissue characteristics and neovascularization using SR-based X-ray in-line phase contrast computed tomography (IL-XPCT) without any contrast agent.

**Results:**

The effect of the energy level and object–to-detector distance on phase-contrast difference was in good agreement with the theory of IL-PCI. Boundaries between the tumor and adjacent normal tissues at week 1 were clearly observed in two-dimensional phase contrast projection imaging. A quantitative contrast difference was observed from weeks 1 to 4. Moreover, 3D image reconstruction of hepatocellular carcinoma (HCC) samples showed blood vessels inside the tumor were abnormal. The smallest blood vessels measured approximately 20 μm in diameter. The tumor vascular density initially increased and then decreased gradually over time. The maximum tumor vascular density was 4.29% at week 2.

**Conclusion:**

IL-XPCT successfully acquired images of neovascularization in HCC xenografts in nude mice.

## Background

Neovascularization is an important feature of solid tumors [1]. Evaluation of tumor neovascularization is helpful for tumor diagnosis, prognosis and assessment of anti-angiogenic efficacy. Vascular imaging techniques including computed tomography angiography (CTA), magnetic resonance angiography (MRA) and digital subtraction angiography (DSA) are based on the differences in the vascular structures and blood flow between tumor and normal vessels, and have been used to monitor tumor angiogenesis or determine the efficacy of anti-angiogenic therapies. However the spatial resolution, especially when detecting small vessels with a diameter of less than 200 μm is still limited [2, 3]. Even micro-CTA, which has the highest resolution among these methods, can only observe vessels of no less than 50 μm in diameter [4, 5].

Synchrotron radiation (SR) microvascular angiography combined with high-resolution and high-speed imaging systems provide an effective approach to study tumor angiogenesis. In vitro studies at the SPring-8 BL20B2 facility in Japan, using barium sulfate as a contrast agent, have revealed the micro-vessel architecture of VX2 carcinoma specimens [6]. Using iodine as contrast agent, SR micro-angiography reliably detects tumor micro-vessel density in vivo [7]. Neovascularization in Lewis lung cancer tumor located deep inside the body was observed using a three-dimensional reconstruction of micro-CT imaging with barium sulfate as contrast agent [8]. However, all these studies used absorption contrast between the contrast medium and surrounding tissues.

Different from these attenuation-based X-ray imaging, X-ray phase-contrast imaging (PCI) exploits differences in the refractive index of different materials to differentiate structures [9]. Thus, PCI can clearly define weakly X-ray-absorptive biological soft tissues without the use of a contrast agent. Several PCI modalities have been developed, such as interferometry [10], diffraction enhanced imaging (DEI) [11, 12], grating-based phase-contrast X-ray imaging (GB-PCI) [13], and in-line phase contrast imaging (IL-PCI) [14, 15]. Among them, IL-PCI has no requirements of superior temporal coherence within the X-ray source and complex experimental apparatus in the light path. Synchrotron-based X-ray Tomographic Microscopy (SRXTM) has been described as a powerful technique for non-destructive high-resolution investigations of various materials, allowing micrometer and sub-micrometer, quantitative, three-dimensional imaging; other techniques include the Swiss Light Source TOMCAT, a new beamline for Tomographic Microscopy and Coherent radiology experiments, which offers sensitivity to density differentials within soft tissues and permits the accommodation of larger tissue sizes [16, 17]. Although studies are currently limited to the stage of technique optimization with in vitro specimen analysis, SR-based X-ray in-line phase contrast computed tomography (IL-XPCT) has great potential for future clinical diagnostic application.

Using the third-generation synchrotron radiation light source at the Shanghai Synchrotron Radiation Facility (SSRF), IL-XPCT has achieved good results in microvascular and tumor angiogenesis [18, 19]. The aim of the present study was to investigate the morphological characteristics of tumor neovascularization in a human hepatocellular carcinoma (HCC) xenograft model using SR-based IL-XPCT.

## Methods

### Animals

Male BALB/c athymic nude mice (5–6 weeks old, weighing 15–18 g) were purchased from SLAC Laboratory Animal Co., Ltd. (Shanghai, China), and maintained under specific pathogen-free (SPF) conditions at the Animal Center of the Liver Cancer Institute of Zhongshan Hospital affiliated to Fudan University (Shanghai, China). All animal experiments were approved by the Animal Care and Use Committee of Zhongshan Hospital, and were conducted in accordance with all state regulations.

### Cell culture

The HCCLM3 cell line was established by the Liver Cancer Institute, Zhongshan Hospital, Fudan University, China, as a HCC cell line with high metastatic potential. Cells were maintained in high-glucose Dulbecco’s modified eagle medium (D-MEM; GibcoBRL, Grand Island, New York, USA) supplemented with 10% fetal bovine serum (Hyclone, Utah, USA) in a humidified 5% CO_2_ atmosphere at 37 °C.

### Orthotopic xenograft model

A single mouse was subcutaneously injected with 1 × 10^7^/0.2 ml HCCLM3 cells in the right upper flank region for the establishment of a subcutaneous xenograft model. When the subcutaneous tumor reached 1 cm in diameter (approximately 4 weeks after injection), it was removed, cut into small pieces of equal volume (1 mm^3^), and transplanted into the left lobe of the liver of 24 nude mice to establish orthotopic xenograft models, as previously described [20].

### Preparation of liver samples

Each week after grafting, six nude mice were anesthetized by intraperitoneal injection of sodium pentobarbital (0.016 g/mL, 0.5 mL/100 g). After opening the abdominal cavity, a PE 10 catheter (Smiths Medical, London, UK) was inserted into the inferior vena cava to inject heparinized saline. When the liver looked pale, the blood vessels and bile ducts were ligated, and the liver was resected. Specimens were immersed in 4% formaldehyde for tissue fixation at room temperature overnight. The next day, three samples were washed and dehydrated using graded ethanol for IL-PCI. Three other samples were used for immunohistochemistry.

### X-ray IL-PCI settings

**Fig. 1:**
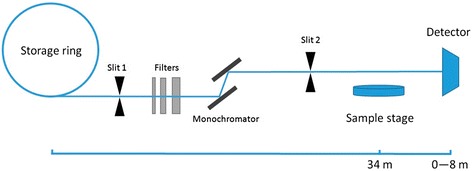
The SSRF BL13W1 imaging device schematic diagram

Neovascularization imaging of tumor xenografts was performed at the X-ray imaging and biomedical application beamline (BL13W1) of the SSRF. The experimental set-up is shown in Fig. 1. The SSRF BL13W1 imaging device was a third-generation synchrotron source with a 200 mA beam current and 3.5 GeV storage energy. The X-ray flux of BL13W1 was several orders of magnitude of X-ray tube flux; the device was designed to provide photon energy ranging from 8 to 72.5 keV with a beam size of 48 mm (horizontal) × 5 mm (vertical) at the object position at 20 keV. Objects are placed at approximately 34 m from the source (storage ring), and the detector can be placed at 0 to 8 m from the objects. Based on the sample size, a high resolution detector VHR 1:1 (Photonic Science, Roberts Bridge, East Sussex, UK) was used with an effective pixel size of 9 μm. Because energy, distance and image quality are not linearly correlated, we used different X-ray energies (12, 15 and 20 keV) and object-to-detector distances (0.05, 1, 3 and 5 m). The optimal X-ray energy and object-to-detector distance were selected and used in subsequent experiments.

For the acquisition of CT images, the sample was rotated 180° at a speed of 0.25°/s, for a total of 1200 projection images. The exposure time of each projection image was 2s. All projection images were transformed into digital slice sections using the fast slice reconstruction software (compiled by the BL13W1 experimental station) based on the filtered back projection (FBP) algorithm. Three-dimensional reconstruction was obtained using the VG Studio Max 3D reconstruction software (version 2.1, Volume Graphics GmbH, Germany).

## Image analysis

### Phase contrast image evaluation

**Fig. 2:**
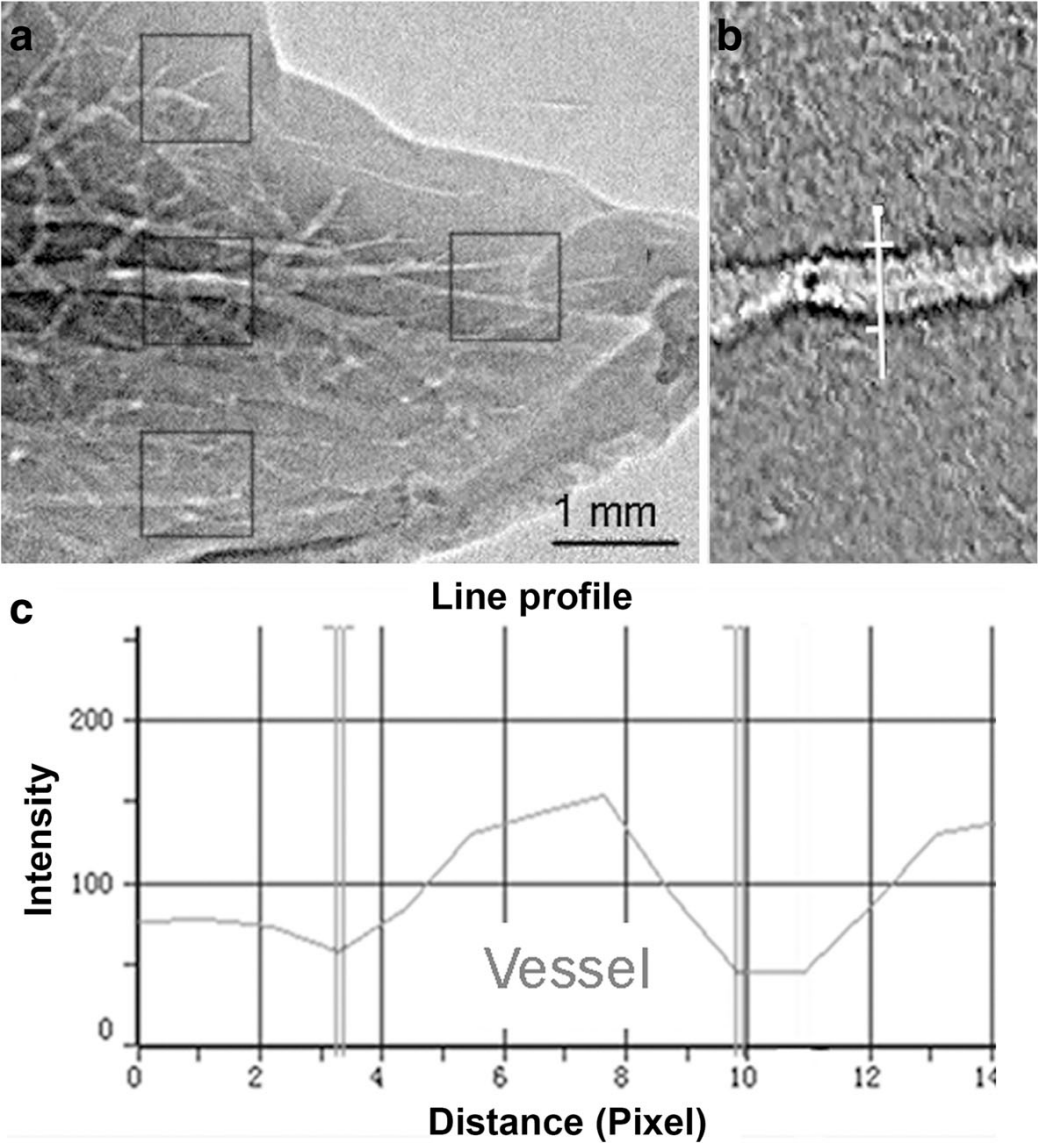
Method for calculating image contrast. **a** Four ROIs were labeled, and the two internal vessels were selected for calculating image contrast. Vessel boundaries were identified using a computer software (Image Pro Plus 6.0) that can identify both edges of a vessel (**b**) by expressing a density curve with a 256 gray-scale image (**c**)

A normal hepatic lobe was taken for imaging at different X-ray energy levels and object-to-detector distances. Four regions of interest (ROIs) and two internal vessels (Fig. 2a) were selected in each frame to calculate the image contrast according to the following formula [8, 21]:

1 $$ C=\frac{I_{max}\mathit{\hbox{-}}{I}_{min}}{I_{max}+{I}_{min}} $$

in which *I*_*max*_ and* I*_*min*_ represent the gray scale values on either side of the blood vessel wall as determined using the Image Pro Plus 6.0 software (Media Cybernetics Inc., Rockville, MD, USA; Fig. 2b, c).

### Quantification of tumor neovascularization using two-dimensional phase-contrast projection

Compared with surrounding normal liver tissues, the tumor vessels are relatively smaller, with a low density, resulting in different vascular boundary enhancements in two-dimensional projection images. Based on this principle, a two-dimensional phase-contrast image projection at different tumor growth stages was analyzed using the Analyze 10.0 software (Analyze Direct, Inc., Lenexa, KS, USA). First, a threshold method was used to correct the area with transmittance below a given value to eliminate the impact of the suture inside the tumor. To quantify the fluctuation of image intensities formed in the detector plane, we used the following equation to calculate the contrast projection, which is composed of overlapping local contrasts [22]:

2 $$ C\left( x, y\right)=\frac{\sqrt{< I{\left( x, y\right)}^2{>}_W-< I\left( x, y\right){>}_W^2}}{< I\left( x, y\right){>}_W} $$

In this formula, *(x, y)* represents a point in the image, *I *is the intensity; subscript *W* denotes the size of the local calculation window, and the operator < * > signifies averaging over the local window.

### Quantitative analysis of tumor neovascularization using SR-based IL-XPCT

Tomographic images at different tumor growth stages were analyzed using the Analyze 10.0 software. The tumor was segmented manually in the tomographic image to calculate tumor volume (mm^3^). A threshold method was used to extract vessels from large quantities of data. The smallest tumor vascular diameter (μm), tumor neovascularization volume (mm^3^) and vascular density (tumor neovascularization volume/tumor volume) were assessed [23].

### Immunohistochemistry analysis of microvessel density

Tumor specimens were fixed in 4% formalin, embedded in paraffin, and sliced into 4-μm-thick serial sections. Slices were analyzed by hematoxylin-eosin (H&E) staining. Microvessel density (MVD) was determined by immunohistochemistry using an anti-CD34 antibody (C-18, Santa Cruz Biotechnology Inc., USA). The number of vessels was scored using a previously described method [24] under a light microscope at 200 × magnification. Any single or cluster of cells with brown staining and clearly separated from adjacent microvessels, tumor cells, and other connective-tissue elements were counted as a single microvessel.

### Statistical analysis

Quantitative data were presented as mean ± standard deviation (SD), except for MVD (non-normal distribution), which was expressed as median (interquartile range [IQR]). Normally distributed data were analyzed by repeated measures analysis of variance (ANOVA), with post hoc Bonferroni t-tests. Simple linear regression models were used to assess the trend of the changes of tumor development by analyzing tumor- and vascular volumes (mm^3^) in relation to time. For MVD, differences between samples were analyzed using the Kruskal-Wallis test. All *P*-values were two sided, and *P* < 0.05 was considered statistically significant. Data were analyzed using SAS 9.2 (SAS Institute, Inc., Cary, NY, USA).

## Results

### Selection of imaging conditions

**Fig. 3:**
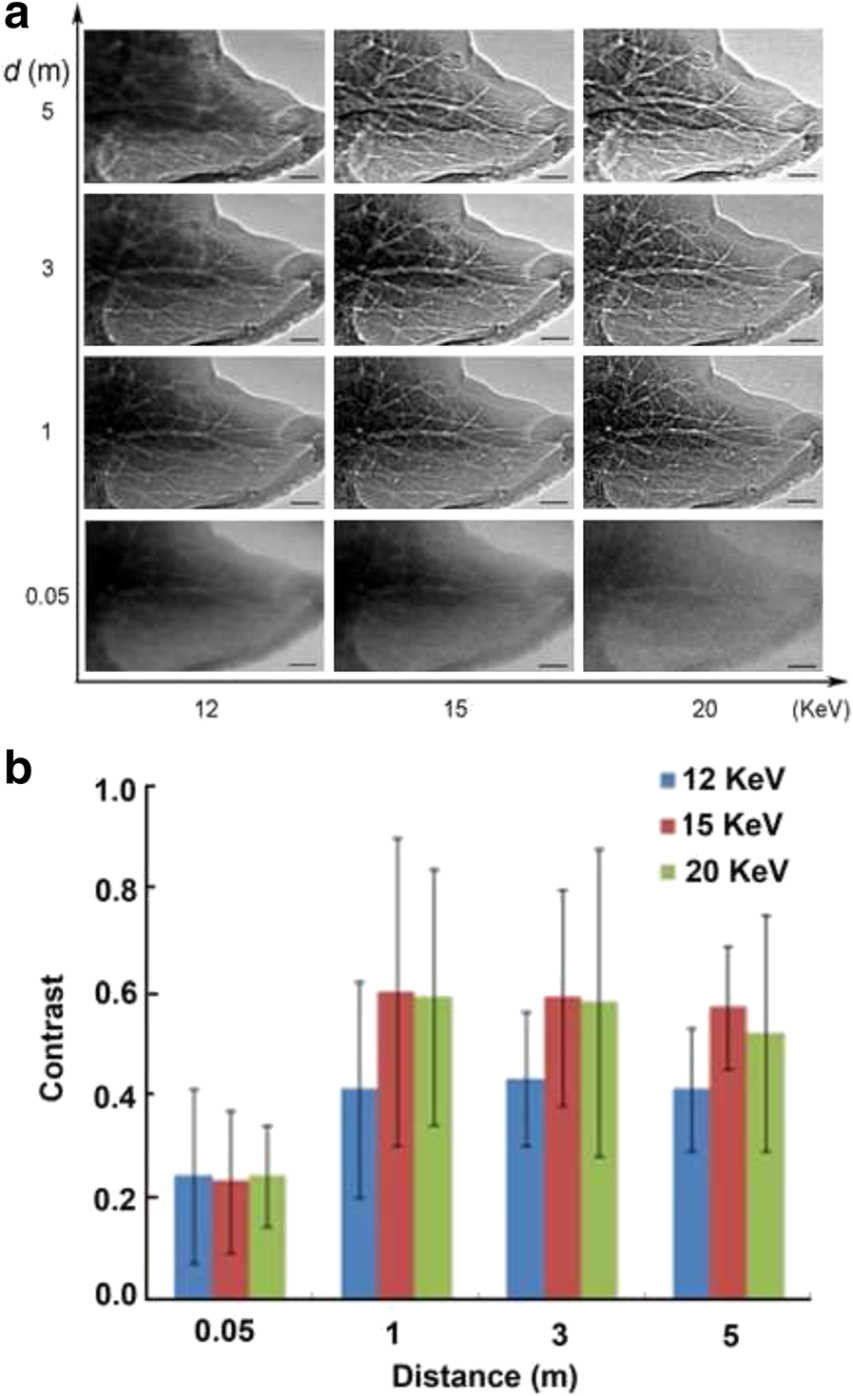
Two-dimensional projection images vs. hepatic vascular phase contrast at different X-ray energies and object-to-detector distances. **a** Normal liver lobe with X-rays set to 12, 15 and 20 keV. Each energy condition was used at 0.05, 1, 3 and 5 m. Bar = 1 mm. **b** Quantitative comparison of image contrast at different X-ray energy levels and object-to-detector distances. The best image contrast was obtained using 15 keV and at 1 m

We first tested result quality using a normal liver sample. Figure 3a shows a normal liver lobe imaged at different X-ray energy levels and object-to-detector distances. Using an object-to-detector distance of 0.05 m, only large branching vessels were visible with low contrast, and the liver boundaries were obscure. At 15 keV, when the distance was increased to 1 m, the progressive branching of the liver vessels was visible, and the tiny vessels at the outer edge of the liver lobe were clearly displayed. However, images were slightly blurry when the distance was increased from 3 to 5 m. Therefore, the X-ray energy was set to 15 keV and the object-to-detector distance at 1 m for the subsequent experiments (Fig. 3b).

### Tumor neovascularization using X-ray IL-PCI

**Fig. 4:**
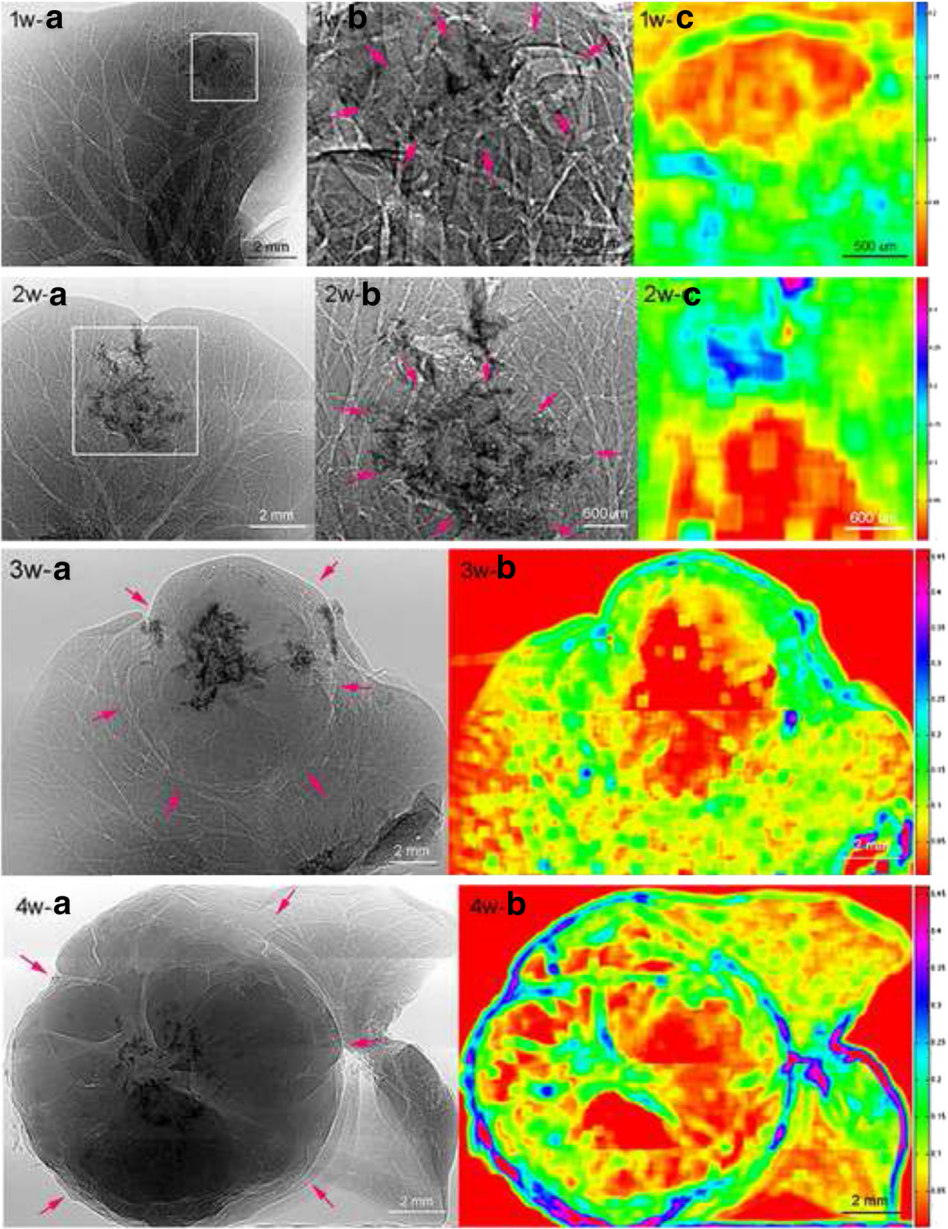
Two-dimensional phase contrast projection imaging of HCCLM3 liver xenografts at weeks 1 to 4. 1w-b and 2w-b are magnified images of the boxed regions in 1w-a and 2w-a, respectively. The red arrows indicate the margins of the tumor, in which the normal hepatic vascular structure was damaged and replaced. The dark region is the shadow from the suture. The vascular boundary enhancement in the tumor region was detected as low contrast in the quantitative analysis results (1w-c, 2w-c, 3w-b, 4w-b). Normal liver tissue presented as a thin edge with low contrast, close to the tumor tissue (3w-b, 4w-b)

The two-dimensional phase contrast projection imaging showed that the normal liver vascular structures were occupied by the tumor tissue (Fig. 4, 1w-a, 1w-b, 2w-a, 2w-b, 3w-a and 4w-a), with clear boundaries between tumor and non-tumor tissues. Along with an increased tumor volume, the tumor margin and peripheral vasculature were under increased pressure, showing tissue compression. The distribution of blood vessels within the tumor was disorganized and had an irregular appearance. In addition, the tumor presented a lobulated shape as the tumor volume increased.

Quantification analysis of the X-ray phase contrast images (Fig. 4, 1w-a, 1w-b, 2w-a, 2w-b, 3w-a and 4w-a) using Eq. (2) showed significant differences in vascular boundary enhancement effects at weeks 1 to 4, which are represented by a higher contrast in the normal liver tissue, and a relatively low contrast in tumor tissue (Fig. 4,1w-c, 2w-c, 3w-b and 4w-b). Along with increasing tumor volume, a large number of vessels at the tumor margin were compressed. The vascular boundary enhancement resulted in higher contrast (Fig. 4,4w-b). It is noteworthy that the normal liver tissue presented as a thin edge. Vascular boundary enhancement was lower in these regions compared with thicker liver tissues near the porta hepatis or close to liver tumor tissues, resulting in lower contrast (Fig. 4,3w-b and 4w-b).

**Fig. 5:**
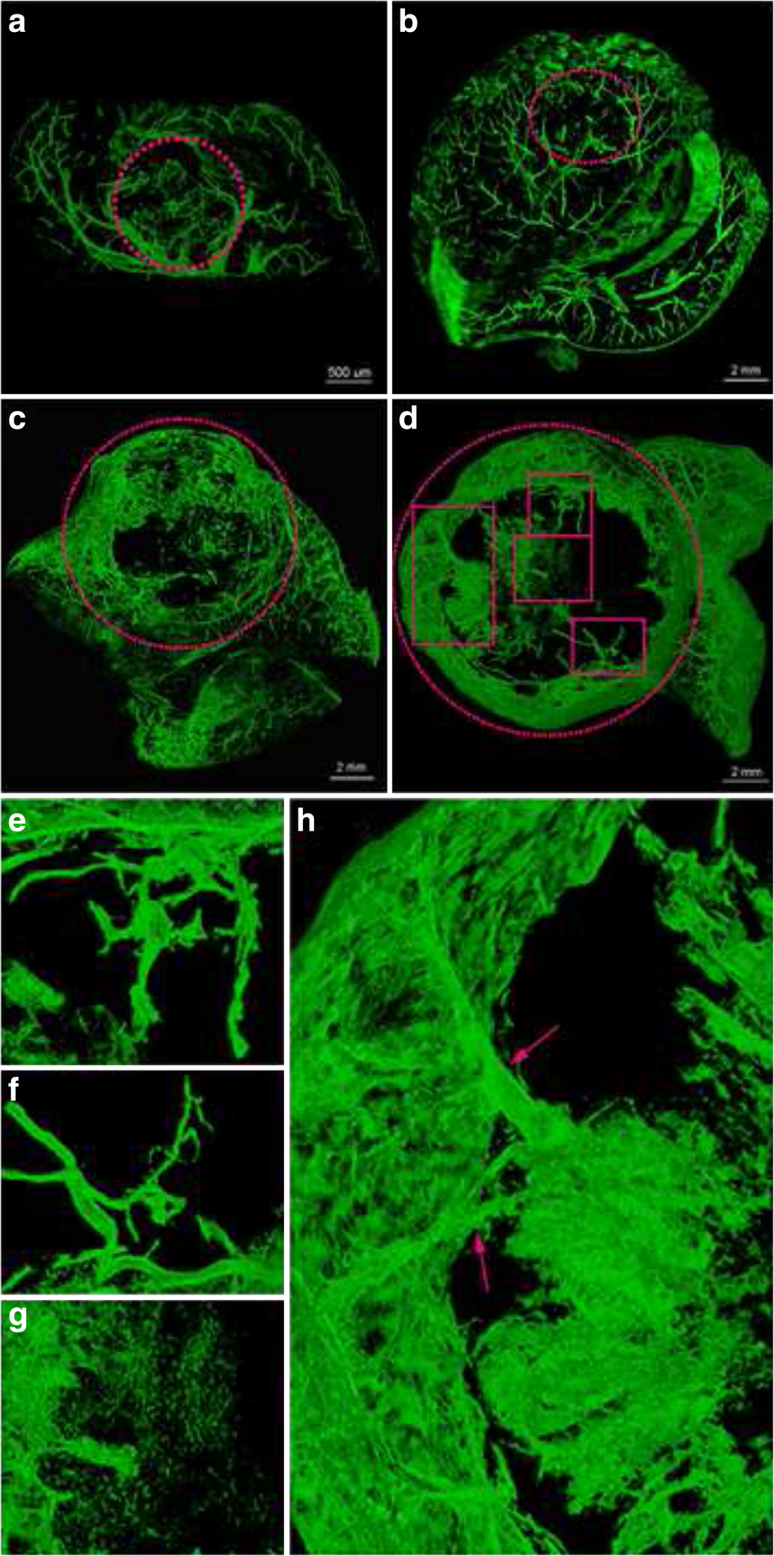
3D reconstruction of contrast CT images for HCC neovascularization at weeks 1 to 4 (**a** to **d**, respectively). The selected areas with red dot lines show the tumor regions, (**e** to **f**) enlarged image of the rectangle region of d, which clearly shows the disorganized distribution and morphology of tumor neovascularization. A large number of avascular regions were observed in the tumors at weeks 3 and 4 (**c**, **d**), as well as irregular vessel shape with dendritic-like branching (**e**), individual vascular curvature abnormalities (**f**), blood vessel network cluster structure (**g**), a large number of tiny and curved vessels derived from a few thick vessels (**h**, *red arrows*), and compressed tumor edge or peripheral vasculature (**c**, **d**)

Figure 5 presents a three-dimensional structural reconstruction of tumors at different growth stages. There was much neovascularization at weeks 1 and 2 (Fig. 5a and b), but the avascular regions gradually increased thereafter (Fig. 5c and d). Vessels had an irregular shape with partially visible dendritic branching (Fig. 5e). There were abnormal curvatures of individual vessels, with both large and small curvatures (Fig. 5f). A vessel network cluster structure was seen within the tumor at weeks 3 and 4 (Fig. 5g). A large number of curved tiny blood vessels branched from several large vessels (Fig. 5h). Finally, tumor edge or peripheral vessels were compressed, and presented as having arcuate displacement (Fig. 5c, d).

Table 1 shows tumor volume, vascular volume and vascular density at weeks 1 to 4. Tumor volume and vascular volume increased with time, but the changes in tumor volume were much greater than those in tumor vascular volume. Although the number of new vessels increased gradually, the tumor growth rate was greater than the angiogenetic. Therefore, vascular density first increased and then decreased during growth. At week 2, tumors had the highest vascular density 4.29 ± 0.49%. The smallest blood vessels measured in SR images were approximately 20 μm in diameter. At different stages of tumor growth, vessels of 27 to 54 μm in diameter had the highest density.

**Table 1:** Characteristics of the HCCLM3 liver xenografts at different time points

	n	Time points				Linear regression coefficient	*P*-value
		Week 1	Week 2	Week 3	Week 4		
Tumor volume (mm^3^)	3	3.78 ± 0.44	27.97 ± 7.33	129.27 ± 23.06	409.10 ± 33.71	0.91993	<0.001
Vascular volume (mm^3^)	3	0.13 ± 0.02	1.18 ± 0.23	3.33 ± 0.25	6.17 ± 0.35	0.97726	<0.001
Vascular density (%)	3	3.51 ± 0.14	4.29 ± 0.49	2.62 ± 0.29	1.52 ± 0.15	—	0.5784

Data are presented as mean ± standard deviation (SD)

**Fig. 6:**
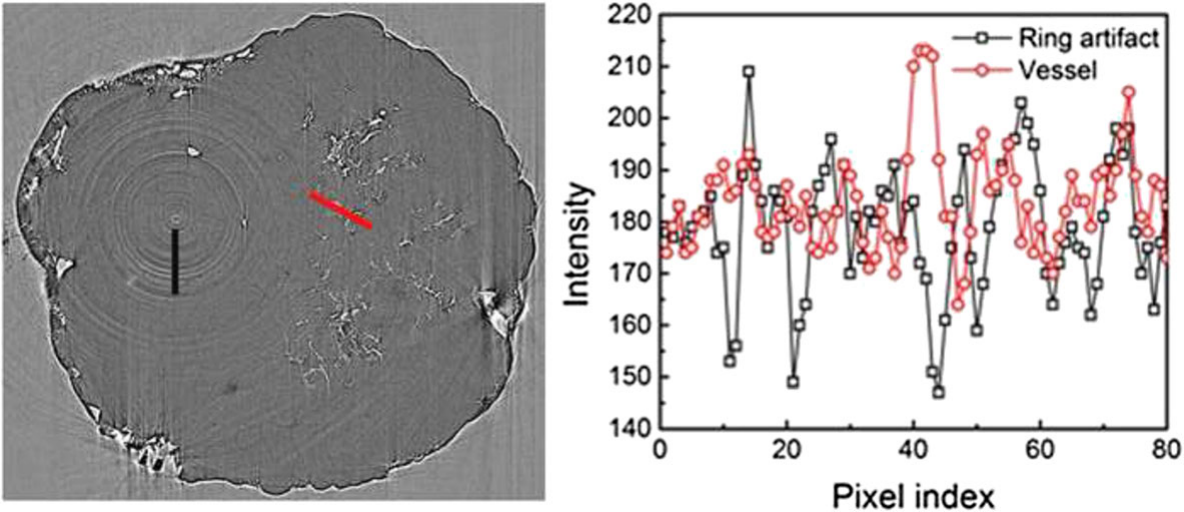
Gray scale analysis of vessels and ring artifacts using two-dimensional PCI tomographic imaging. *Left*: The *gray* scale analytical measurement ranges of vessels and ring artifacts is indicated in *red* and *black. Right*: The vertical axis in the graph is *gray* intensity, the abscissa is pixel number, labeling the corresponding pixel position of each *gray* value. *Gray* scale values between vessels and ring artifacts were similar, with no statistically significant differences

We used gray scale analysis to monitor the influence of ring artifact (Fig. 6). The gray intensity difference between the vessels and ring artifacts were close, and there were no significant differences in phase contrast CT. Therefore, the ring artifacts had a significant impact on vascular identification during the vessel extraction process.

### Micro-vessel density by immunohistochemistry

**Fig. 7:**
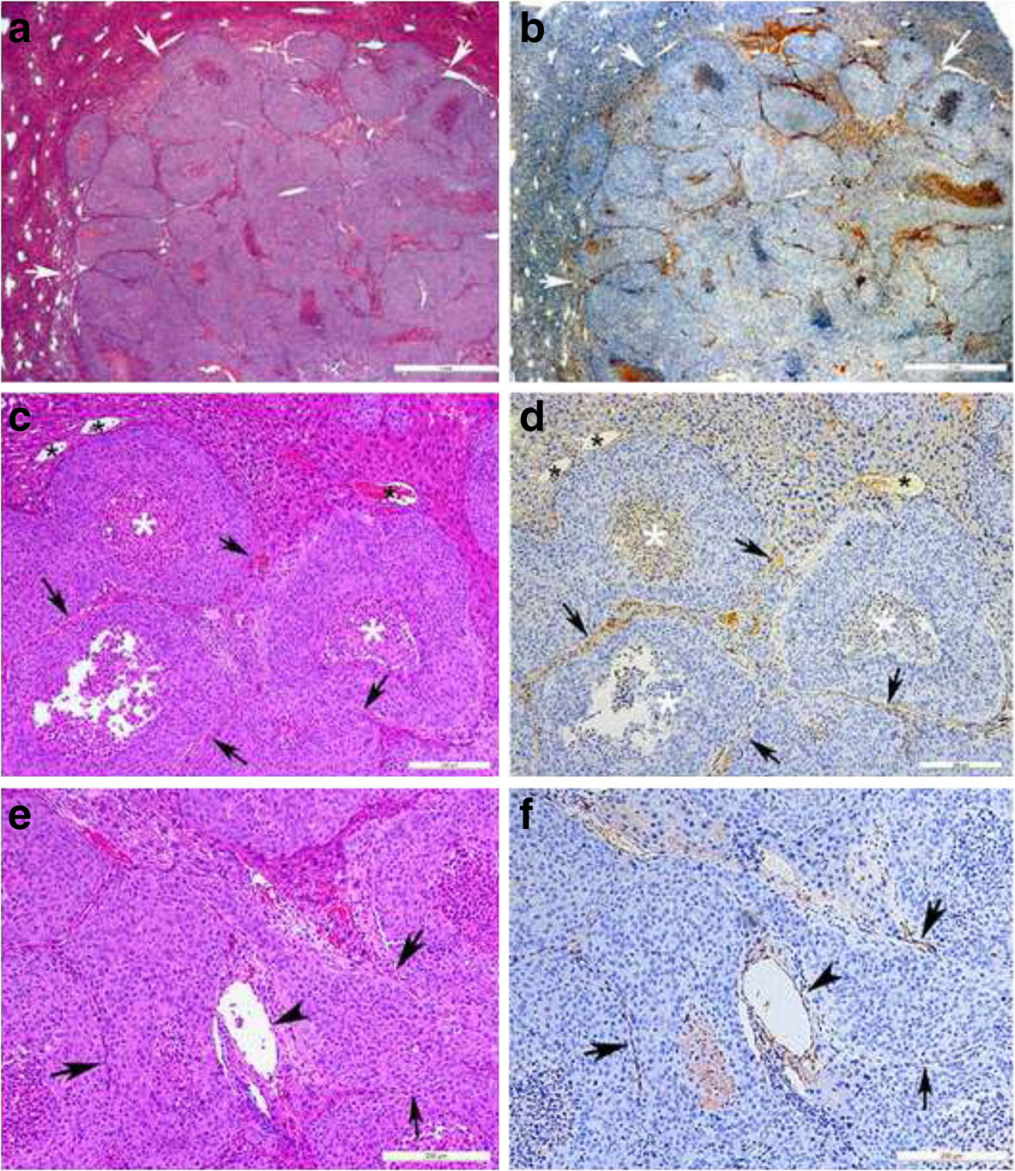
Histopathological images of HCCLM3 orthotopic xenograft tumors at 2 weeks. *Left*: H&E staining. *Right*: CD34 immunohistochemistry. **a b** Histopathological images of cancer tissues at week 2. Scale bar = 1 mm. **c d** Histopathological images of cancer tissues at week 2. Bar = 200 μm. **e f** Histopathological images of cancer tissues at week 3. Bar = 200 μm. The boundaries between tumor and normal liver tissue could be shown clearly (**a** and **b**, *white arrow*). CD34 expression was positive in the abundant normal hepatic sinusoid (**c** and **d**, *black asterisks*) and tumor angiogenesis (**c** to **f**, *black arrows* and *arrow heads*). The tumor edge or peripheral vasculature was compressed, having a shift in its curvature. The microvascular-rich regions were diffusely distributed at the edge of tumor nests. New vessels with different diameters were seen. The abnormal large vessels (**e** and **f**, *black arrow heads*) and slit-like small vessels (**c** to **f**, *black arrows*) coexisted. The characteristics of the vascular arrangements in PCI images were partly similar with those of histological sections. In addition, necrosis could be seen in the tumor nest (**c** and **d**, *white asterisks*). The MVD of CD34 expression in tumors was determined by immunohistochemistry. Data are shown as median (Q25, Q75)

H&E staining and CD34 immunohistochemistry results are shown in Fig. 7. The boundaries between tumor and normal liver tissue could be shown clearly (Fig. 7a and b, white arrows). CD34 expression was positive in the abundant normal hepatic sinusoid (Fig. 7c and d, black asterisks) and tumor angiogenesis (Fig. 7c to f, black arrows and arrow heads). Tumor edge or peripheral vasculature was compressed. Distribution of angiogenesis was disordered within the tumor. The micro vascular-rich regions were diffusely distributed at the edge of tumor nests. Neovascularization with different diameters was seen, and abnormal large vessels (Fig. 7e and f, black arrow heads) and slit-like small vessels (Fig. 7c to f, black arrows) coexisted. The characteristics of vascular arrangements in PCI images were partly similar with that of histological sections. In addition, necrosis could be seen in tumor nests (Fig. 7c and d, white asterisks).

The MVD based on CD34 expression at weeks 1 to 4 was 25 (12–35) vessels/high-powered field (HPF), 16.5 (15–20) vessels/HPF, 29 (16–40) vessels/HPF, and 20 (15–25) vessels/HPF, respectively. There were no significant differences in MVD values between time points (*P* = 0.758).

## Discussion

Neovascularization reflects tumor behavior and characteristics, and has received much attention in recent years [25, 26]. PCI displays a high sensitivity for soft tissues such as blood vessels [4, 15, 19, 27, 28]. In the present study, the neovascularization of transplanted HCCLM3 tumors was imaged at different growth stages, and morphology and spatial distribution of tumor angiogenesis were observed.

For IL-PCI, the X-ray energy and the distance from object to detector are two important parameters [8, 9, 29]. Indeed, the lower the energy, the higher the contrast. However, low energy reduces X-ray penetration, extends imaging time, and increases the radiation dose. Thus, the energy level should be selected according to the specific target object in order to balance contrast and radiation dose. Based on Fresnel’s diffraction theory of wave optics, the object-to-detector distance is zero in traditional absorption imaging. However, phase-contrast images are formed with greater distances from the object. The visual appearance of phase-contrast enhancement in the final image is the edge enhancement at interfaces between components with differing X-ray refraction indices. Increasing object-to-detector distance leads to phase contrast enhancement, which is affected by the interference between the sample and X-rays; however, a distance beyond a certain range causes decreased spatial resolution, with the image losing resemblance [9]. Also, PCI details increase with time, because prolonged exposure to air and heat from the X-rays gradually dehydrates the samples [21, 30]. In addition, specimen thickness, the fixation method, and exposure time are important factors affecting image quality [21, 30]. According to the sample size and thickness, a CCD detector with a resolution of 9 μm was selected, the X-ray energy was set to 15 keV, and the distance from source to detector was 1 m. Based on the principle that the image occupies the whole dynamic range without saturation, we selected the appropriate exposure time and obtained a satisfactory edge enhancement effect using IL-PCI.

Based on the differences in vascular edge enhancement between tumors and adjacent normal liver tissues, quantitative analysis of the X-ray phase contrast images showed a higher contrast in the normal liver tissue, and a relatively lower contrast in tumor tissues. However, the calculation method used in this study is affected by many factors, including tissue thickness and the signal-to-noise ratio [22].

IL-XPCT is suitable for the detailed 3D morphological imaging of small structure [27, 31–33]. As shown above, differences were found in tumor vascular density at different growth stages. Tumor vascular density first increased and then decreased over time. This may be related to the highly malignant ability of the HCCLM3 cell line used in the present study. At the late stage of tumor growth, tumor cells multiply quickly, leading to inadequate nutrient supply and abundant tumor necrosis. In addition, the smallest distinguishable tumor vessel diameter was found to be 20 μm.

Currently, immunohistochemistry is considered the “gold standard” for measuring MVD [24]. However, no significant difference in MVD was found in this study during the four weeks; this may be due to the small sample size. In addition, immunohistochemistry only reveals the two-dimensional distribution of blood vessels, and the observer selectively counts the tumor vessels in the “hot zone”. Therefore, immunohistochemistry may not reflect the morphology and spatial distribution of tumor angiogenesis, resulting in the loss of potentially critical information about the vascular structure.

However, phase-contrast CT imaging is more challenging in tumor angiogenesis imaging and quantitative calculations than absorption-contrast CT. Indeed, phase-contrast CT suffers from ring artifacts: the gray value difference between the vessel and ring artifacts is small, rendering difficult the threshold setting of vascular segmentation in CT images and interfering with blood vessel identification. If the set threshold is too low, ring artifacts may be classified as vessels, thereby over-evaluating the vessel density. If it is too high, actual vessel density will be underestimated. Future studies should be carry out to remove ring artifacts and improve the ability to identify vessels. During tumor growth, both necrotic tissue and tiny vessels were similarly gray. Therefore, it was difficult to distinguish between the two entities. Thus, vascular density in vessel segmentation may be overestimated. Finally, hand-drawing of the tumor region was subjective, as was the selection of the areas by different observers.

The present study had some limitations. First, a 9 μm CCD detector was chosen considering sample size and thickness; therefore, the smallest vessel diameter detected was approximately 20 μm. Since the capillary diameter varies from a few to a hundred μm, vessels of less than 20 μm were not observed and tumor vascular density could be underestimated. Therefore, we will use higher-resolution detectors in future studies. Second, the X-ray energy and object-to-detector distance were selected based on in vitro experiments. Finally, the imaging results were acquired from ethanol-dehydrated, formalin-fixed specimens. As a pioneer imaging technique, SR-based IL-XPCT, mainly limited by small beam size and high radiation dose, still needs further optimization to suit clinical application. Even in vivo imaging of neovascularization remains a challenging task. Therefore, the currently available studies mainly concentrate on in vitro specimen imaging to optimize the settings as well as to find the potential application field. In the future, provided that the problems related to technical facilities, imaging parameters and experimental model preparation are resolved, in vivo application of IL-XPCT is promising. Optionally, combining GB-PCI with conventional X-ray tube may be applicable in clinical conditions. Future studies should also examine bone samples from bone tumors or metastatic bone lesions using IL-XPCT, comparing the results with existing micro-CT data for such specimens.

## Conclusion

In this study, we demonstrated that SR-based IL-XPCT successfully acquires images of neovascularization in liver tumor xenografts. This technique can distinguish tumors from the normal liver tissue, and detect blood vessels as small as 20 μm in diameter. In addition, quantitative analysis showed that vascular density increases first and then decreases gradually with tumor growth.

## Abbreviations

CTA: Computed tomography angiography
DEI: Diffraction enhanced imaging
DSA: Digital subtraction angiography
GB-PCI: Grating-based phase-contrast X-ray imaging
IL-PCI: In-line phase contrast imaging
IL-XPCT: In-line X-ray phase contrast computed tomography
MRA: Magnetic resonance angiography
PCI: Phase-contrast imaging
SR: Synchrotron radiation
SRXTM: Synchrotron-based X-ray tomographic microscopy
SSRF: Shanghai synchrotron radiation facility
